# Identification of Radiation-Induced Injury Pathways and Hub Genes from RNA-Seq Data Based on Integrative Bioinformatics Approach

**DOI:** 10.3390/genes17040377

**Published:** 2026-03-27

**Authors:** Khalish Arsy Al Khairy Siregar, Chi-Ho Lee, Jong-Jin Kim, Dong-Jo Chang, Seung-Hyun Jeong

**Affiliations:** 1 College of Pharmacy, Sunchon National University, 255 Jungang-ro, Suncheon-si 57922, Jeollanam-do, Republic of Korea; 1250267@s.scnu.ac.kr (K.A.A.K.S.); dlclgh1010@naver.com (C.-H.L.); djchang@scnu.ac.kr (D.-J.C.); 2 Department of Biomedical Science, Sunchon National University, Suncheon-si 57922, Jeollanam-do, Republic of Korea; kimjj@scnu.ac.kr; 3 College of Pharmacy and Research Institute of Life and Pharmaceutical Sciences, Sunchon National University, Suncheon-si 57922, Jeollanam-do, Republic of Korea

**Keywords:** ionizing radiation, bone marrow injury, RNA sequencing, integrative bioinformatics analysis, hub genes

## Abstract

Background: Ionizing radiation (IR) induces profound bone marrow (BM) injury by disrupting hematopoietic stem cell (HSC) homeostasis, leading to acute myelosuppression and long-term hematopoietic dysfunction. Although transcriptome-wide analyses have advanced our understanding of radiation responses, the key molecular networks and hub genes governing post-irradiation BM injury remain incompletely defined. Methods: This study aimed to systematically identify radiation-responsive pathways and central genes in BM after irradiation through an integrative bioinformatics approach based on RNA sequencing (RNA-seq). Public RNA-seq data from mouse BM HSCs collected 3 days after whole-body irradiation were analyzed. Differentially expressed genes (DEGs) were identified using two independent statistical frameworks to improve the robustness of the results. Functional analysis was performed through Gene Ontology (GO), Kyoto Encyclopedia of Genes and Genomes (KEGG), and Gene Set Enrichment Analysis (GSEA). Protein–protein interaction (PPI) networks were constructed using STRING, and hub genes were identified using network topology parameters. Results: Both analysis pathways consistently demonstrated extensive transcriptome reprogramming after irradiation. DEGs were primarily enriched in processes related to cytokine signaling, hematopoietic lineage regulation, immune response, and extracellular matrix remodeling. KEGG analysis highlighted cytokine–cytokine receptor interaction, hematopoietic cell lineage, JAK-STAT signaling, and PI3K-Akt signaling as key molecular axes. GSEA further supported coordinated changes in pathways related to inflammatory response, stress response, and metabolic reprogramming. PPI network analysis identified four consensus hub genes, namely *Il6*, *Cd34*, *Gypa*, and *Pdgfrb*, which are related to inflammatory signaling, hematopoietic regulation, erythroid dynamics, and microenvironmental remodeling, respectively. Conclusion: This integrative bioinformatics study demonstrates that radiation-induced BM injury is associated with coordinated activation of inflammatory cytokine networks, alterations in the hematopoietic program, and microenvironmental restructuring. The hub genes identified in this study may represent candidate regulatory genes or molecular indicators potentially involved in the response to radiation-induced hematopoietic damage.

## 1. Introduction

Ionizing radiation (IR) is widespread in the environment and widely used in medicine and industry [1]. However, IR remains a significant issue in clinical contexts, ranging from side effects of cancer radiotherapy to accidental exposure due to nuclear accidents or industrial incidents [2]. Bone marrow (BM) is one of the organs most vulnerable to IR because it serves as the center of hematopoiesis and relies on a population of hematopoietic cells that are sensitive to genotoxic stress [3,4]. IR exposure can cause acute BM suppression, characterized by decreased leukocytes, platelets, and erythrocytes, increasing the risk of infection, bleeding, and anemia, and contributing to radiation-induced hematopoietic syndrome [5,6]. At the cellular level, IR can damage hematopoietic stem cells (HSCs) and significantly deplete more proliferative hematopoietic progenitor cells (HPCs) and their more mature progeny, disrupting hematopoietic homeostasis and impeding hematologic recovery [7,8].

Currently, clinical management primarily focuses on supportive care and hematopoietic growth factor administration, but their effectiveness is not always sufficient to prevent long-term HSC dysfunction [1]. Although some patients recover relatively quickly from acute myelosuppression following irradiation or chemotherapy, a significant number of cases still experience long-term BM injury characterized by a reduced HSC reserve and impaired HSC self-renewal capacity [9]. This situation emphasizes that recovery of hematological parameters in the early phase does not always reflect full recovery of HSC function. Therefore, identifying effective mitigation strategies and therapeutic targets remains challenging, partly because the molecular and genetic mechanisms underlying IR-induced injury remain incomplete.

In recent years, efforts to identify radiation-responsive genes at the genome-wide scale have intensified. With advances in RNA-sequencing technology, high-resolution transcriptome analysis enables comprehensive mapping of gene expression changes after radiation exposure [10,11]. Therefore, this study performed an integrative computational reanalysis of publicly available RNA-sequencing data from radiation-exposed mouse HSC to identify consensus transcriptional signatures and candidate regulatory genes associated with radiation-induced BM injury. This approach provides an opportunity to more systematically understand the biological responses involved in radiation-induced hematopoietic injury, including cytokine signaling, signal transduction, and hematopoietic regulatory disruption. Furthermore, protein–protein interaction (PPI) network analysis can be used to prioritize hub genes that play important roles in these regulatory networks.

Although the original dataset reported by Liao et al. (2024) [12] primarily highlighted the regulation of HSC regeneration and IGF1-mediated ferroptosis after irradiation, the broader transcriptional network underlying radiation-induced hematopoietic injury remains incompletely characterized. Therefore, this study performed an integrative reanalysis of the dataset by combining differential expression analysis, functional enrichment analysis, and network topology-based identification of hub genes. Within this framework, this study aimed to gain a systems-level perspective on molecular responses in BM after irradiation and to prioritize candidate genes and biological pathways potentially associated with radiation-induced hematopoietic injury.

## 2. Materials and Methods

### 2.1. Data Exploration and Acquisition

Raw RNA-sequencing (RNA-seq) data were obtained from the NCBI Sequence Read Archive (SRA) on BioProject PRJNA1026541 (accessed on 2 October 2025) as reported by Liao et al. (2024) [12]. The datasets used were from two conditions: the total body irradiation (TBI) group of HSCs of mice at 3 days after IR (SRR26338102, SRR26338103, and SRR26338105) and the wild-type control group (SRR26338106, SRR26338107, and SRR26338108). Based on the reference study, mice were exposed to a single dose of 5 Gy TBI. FASTQ files were downloaded using the SRA Toolkit and uniformly processed through a standard bioinformatics workflow that includes sequencing quality control, adapter and low-quality read cleaning when necessary, mapping/quantification against the mouse reference genome, and differential expression analysis to ensure consistency and reproducibility.

### 2.2. Quality Check and Preprocessing Analysis

The RNA-Seq analysis protocol begins with a quality evaluation of raw data to identify low-quality reads that may impact downstream analysis and biological interpretation. Data quality is checked using FastQC [13], which generates an HTML report summarizing various Illumina read-quality metrics, including per-base sequence quality, adapter content, overrepresented sequences, and GC content [3]. Following the initial evaluation, adapter trimming and filtering of low-quality reads are performed using fastp software, version 0.12.1 (https://github.com/OpenGene/fastp, accessed on 20 October 2025) [14]. Based on Jeong et al. (2025) [15], the evaluation criteria included per-base sequence quality, adapter content, overrepresented sequences, and QC content. This preprocessing step ensures that all samples meet acceptable quality standards before proceeding to alignment and other downstream analyses.

### 2.3. Mapping, Alignment, and Gene-Level Quantification

RNA-seq data in FASTQ format that had passed the quality control (QC) stage were then mapped to the *Mus musculus* reference genome (GRCm38/mm10) using HISAT2 via the Galaxy web server (https://usegalaxy.eu, accessed on 20 October 2025), which is one of the most efficient and widely recommended alignment methods for RNA-Seq data [16,17]. HISAT2 offers alignment analysis tailored to map various types of RNA-seq reads. Together, these factors enable HISAT2 to align reads quickly and accurately by using index files generated from the reference genome to map pairs of FASTQ files to the target genome. The alignment process produces an output file in Binary Alignment Map (.bam) format, which is then used for gene expression quantification. Gene-level expression quantification was subsequently performed using featureCounts (version 2.1.1 + galaxy0) in Galaxy, with the built-in mm10 reference annotation and default parameters. This step generated a raw read count matrix with genes as rows and samples as columns [18,19]. No explicit low-expression gene filtering was applied prior to differential expression analysis. The resulting raw count matrix was then used as input for downstream differential expression analysis using limma-voom and DESeq2.

### 2.4. Differential Expression Analysis

Differential expression analysis was performed to identify genes showing significant expression changes between the control and IR groups. The raw read count matrix obtained from featureCounts was used as input for the analysis. To improve the reliability and robustness of the results, two complementary approaches, namely limma-voom and DESeq2, were applied via the Galaxy web server. In the limma-voom approach, models were constructed based on treatment condition, with the contrast tested being irradiated vs. control [20]. Both methods were run using their default settings in Galaxy [20,21,22,23]. Genes were defined as differentially expressed genes (DEGs) if they met the criteria of adjusted *p*-value < 0.05, which was corrected using the Benjamini–Hochberg method, and |log2 fold change (FC)| ≥ 2. Genes with log_2_FC ≥ 2 were categorized as genes with increased expression, while genes with log_2_FC ≤ −2 were categorized as genes with decreased expression. The set of significant DEGs generated by each approach was then used for further analysis. For sample-level exploratory analyses, like principal component analysis (PCA) and sample-distance heatmap generation, the normalized/transformed expression matrix generated within the DESeq2 visualization workflow in Galaxy was used.

### 2.5. Pathway Enrichment Analysis

To investigate the biological functions and molecular pathways involved, Gene Ontology (GO) annotation and Kyoto Encyclopedia of Genes and Genomes (KEGG) enrichment analysis were performed using the clusterProfiler package in R software (version 4.5.1), with the reference species org.Mm.eg.db [24]. GO annotations were classified into three main domains, namely Biological Process (BP), Cellular Component (CC), and Molecular Function (MF). A *p*-value and *q*-value < 0.05 were used as the significance threshold to identify significantly enriched GO terms and KEGG pathways [25]. Additionally, to gain a more comprehensive understanding at the whole-transcriptome level, pre-ranked Gene Set Enrichment Analysis (GSEA) was performed using the fgsea package [26]. Genes were ranked based on Wald statistic values from the DESeq2 analysis [27], allowing the direction and strength of expression changes to be considered simultaneously. Hallmark gene sets were downloaded from the Molecular Signatures Database (MSigDB). Significantly enriched pathways were determined based on a *p*-value < 0.05 and an FDR < 0.05, as defined by a predetermined threshold.

### 2.6. Hub Gene Expression Analysis

To identify hub genes from each DEG’s results (limma-voom and DESeq2), a PPI network was constructed using the STRING (https://string-db.org/) database (version 12.0) with *Mus musculus* as the organism and a high confidence threshold (>0.700). All interactions that met these criteria were downloaded from STRING and then imported for further network analysis using Cytoscape (version 3.10.3) [28,29]. Identification of central genes in the network was performed using the CytoNCA plugin by calculating network topology parameters, including degree, betweenness, and closeness centrality [30]. Genes with values above the median for each parameter were selected as candidate hub genes, and these candidates were used to construct a more focused core subnetwork for further analysis [31]. In addition, to improve the reliability and robustness of hub gene identification results and reduce potential bias introduced by a single threshold and a single topology approach, an additional analysis was performed using the CytoHubba plugin; based on the approach reported by Vaghasia H et al. (2022) [32], CytoHubba was used to evaluate node rankings through various topology algorithms, including local and global methods, namely degree, edge percolated component (EPC), maximum neighborhood component (MNC), density of maximum neighborhood component (DMNC), maximal clique centrality (MCC), bottleneck, eccentricity, closeness, radiality, betweenness, stress, and clustering coefficient. Genes consistently identified across multiple algorithms were considered as more robust hub gene candidates. This approach was used to ensure that genes identified by the CytoNCA analysis remained consistent across different topological algorithms.

## 3. Results

### 3.1. Quality Control and Alignment Results

HTML quality control reports were generated using FastQC software (version 0.12.1) for all six RNA-Seq samples downloaded from the NCBI database. Quality evaluation was performed according to the study by Jeong et al. (2025) [15], focusing on key metrics including per-base sequence quality, adapter content, overrepresented sequences, and GC content. Overall, all samples exhibited good read quality, indicated by Phred scores > Q30 at most base positions, consistent GC content (48.57–49.76%), and low levels of overrepresented sequences and adapter contamination after trimming with fastp (Table S1). Next, high-quality reads were aligned using HISAT2, and summary alignment findings for each sample are presented in Table 1.

### 3.2. Identification of Differential Expression Analysis

**Figure 1:**
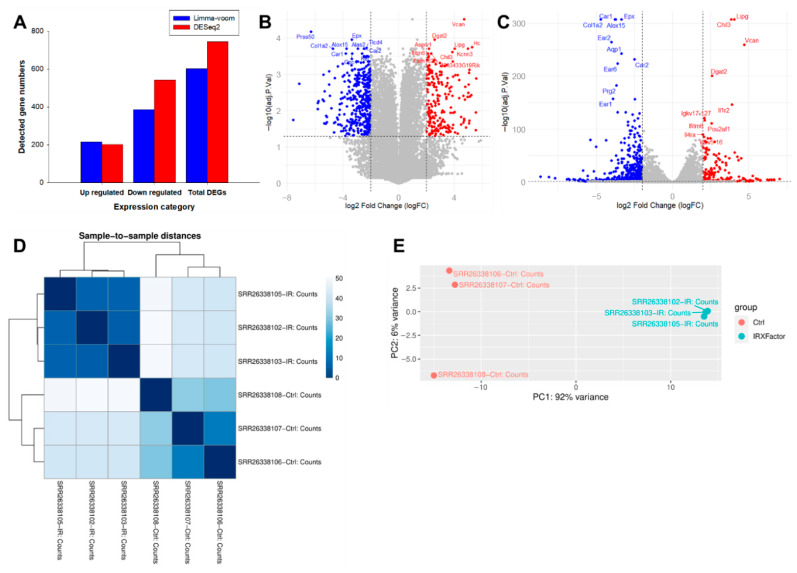
Overview of differential expression analysis and sample-level transcriptomic separation following irradiation. (**A**) Bar plot summarizing the numbers of DEGs identified by limma-voom (blue) and DESeq2 (red) under the criteria of padj < 0.05 and |log_2_FC| ≥ 2, including upregulated, downregulated, and total DEGs. (**B**,**C**) Volcano plots illustrating differential gene expression profiles obtained from limma-voom (**B**) and DESeq2 (**C**) analyses. Upregulated genes are shown in red, downregulated genes in blue, and non-significant genes in gray; dashed lines indicate significance thresholds for log_2_FC and padj. Representative highly regulated genes are labeled. (**D**) Heatmap of sample-to-sample distances based on normalized gene expression values, demonstrating clear clustering according to experimental condition (control vs. irradiation). (**E**) Principal component analysis (PCA) plot showing robust separation between control and irradiated BM samples along the first principal component, which accounts for the majority of variance.

To identify transcriptomic changes, differential expression analysis was performed using two independent statistical approaches, limma-voom and DESeq2, run through the useGalaxy web server. Both methods were applied to count data generated by featureCounts, with uniform significance criteria (padj < 0.05 and |log_2_FC| ≥ 2) to define DEGs. Distance analysis between samples revealed consistent clustering by biological condition (Figure 1D), which was confirmed by PCA, with clear separation between control and irradiated samples in the principal components (Figure 1E), and quality control diagnostics from both analytical pipelines are presented in Figure S1.

Overall, limma-voom identified 601 DEGs (216 increased; 385 decreased) (Table S2). In comparison, DESeq2 identified 744 DEGs (202 increased; 542 decreased) (Table S3 and Figure 1A). Volcano plots showed a distribution of significant genes toward both positive and negative changes in both approaches, supporting substantial expression differences between groups (Figure 1B,C). Furthermore, the patterns of log_2_FC change direction and prominent genes were generally consistent with the limma-voom results. The difference in the number of DEGs between these methods is also consistent with a report by Tong, Y. (2021) [33], which showed that DESeq2 tends to identify more differential genes than limma, while differences in “unique genes” between methods often occur among genes near the significance threshold. Thus, the consistency in the direction of change and the main candidate genes in this study support the idea that both methods capture similar key transcriptomic responses to irradiation.

### 3.3. Functional Enrichment Analysis of DEGs

**Figure 2:**
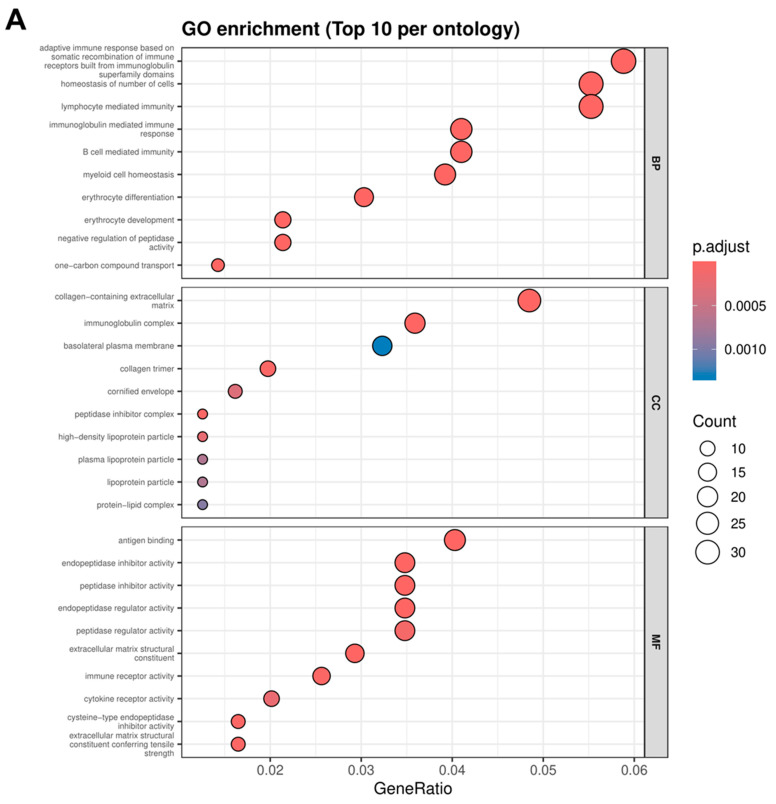

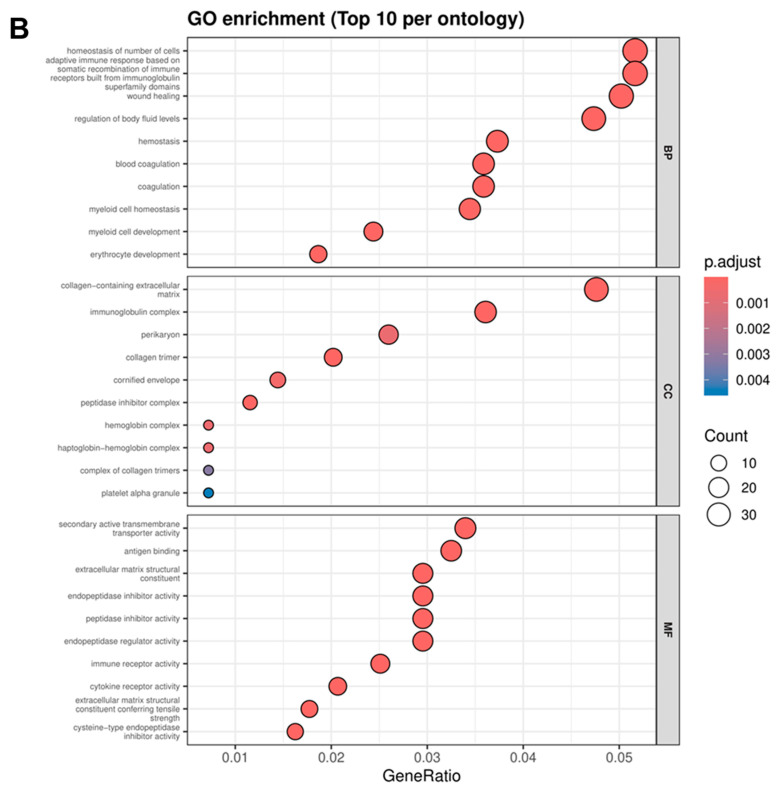
Gene Ontology (GO) enrichment analysis of DEGs following irradiation. (**A**,**B**) Bubble plots showing the top 10 significantly enriched GO terms per ontology category—Biological Process (BP), Cellular Component (CC), and Molecular Function (MF)—based on DEGs identified by limma-voom (**A**) and DESeq2 (**B**). The *x*-axis represents the GeneRatio, and bubble size indicates the number of genes associated with each GO term. Color intensity corresponds to the adjusted *p*-value (padj), reflecting the statistical significance of enrichment. Across both analytical approaches, enriched GO terms consistently highlight immune-related processes, hematopoietic regulation, and extracellular matrix-associated components and functions.

Functional enrichment analysis was performed to understand the biological context of DEGs between the control and radiation-exposed groups. GO analysis, including the categories BP, CC, and MF, showed that DEGs were strongly associated with the adaptive immune response, hemostasis/coagulation processes, and changes in extracellular matrix components (Figure 2 and Table S4). In general, these themes emerged consistently across the results from both DEG analysis approaches (limma-voom and DESeq2), demonstrating the congruence of the biological context captured by both methods. In the BP category, enriched terms included processes related to adaptive immunity and immunoglobulin-mediated immune responses, as well as processes related to myeloid cell homeostasis and erythrocyte development/differentiation. Furthermore, DESeq2 results showed enrichment for terms related to hemostasis and coagulation, as well as processes involved in responses to tissue damage, such as wound healing. Biologically, the enrichment of these processes indicates that radiation exposure is associated with changes in immune and hematopoietic programs, as well as the involvement of homeostasis-maintaining mechanisms common to tissue stress. In the CC category, DEGs were enriched in extracellular matrix components, particularly collagen-containing extracellular matrix and collagen structures, as well as immune-related components such as immunoglobulin complexes. In the DESeq2 results, terms such as platelet alpha granule also appeared, consistent with the enrichment of BPs related to coagulation/hemostasis. This pattern supports changes in the tissue microenvironment (ECM/collagen remodeling) and the involvement of immune components, which are often associated with responses to tissue injury and changes in cell composition after irradiation. In the MF category, enriched terms included antigen binding, immune receptor activity, and cytokine receptor activity, consistent with immune signaling. Furthermore, peptidase inhibitor/regulator functions and ECM-related structural functions also emerged. The enrichment of these functions indicates the involvement of signaling regulation and proteolysis in the biological response to radiation-induced stress.

**Figure 3:**
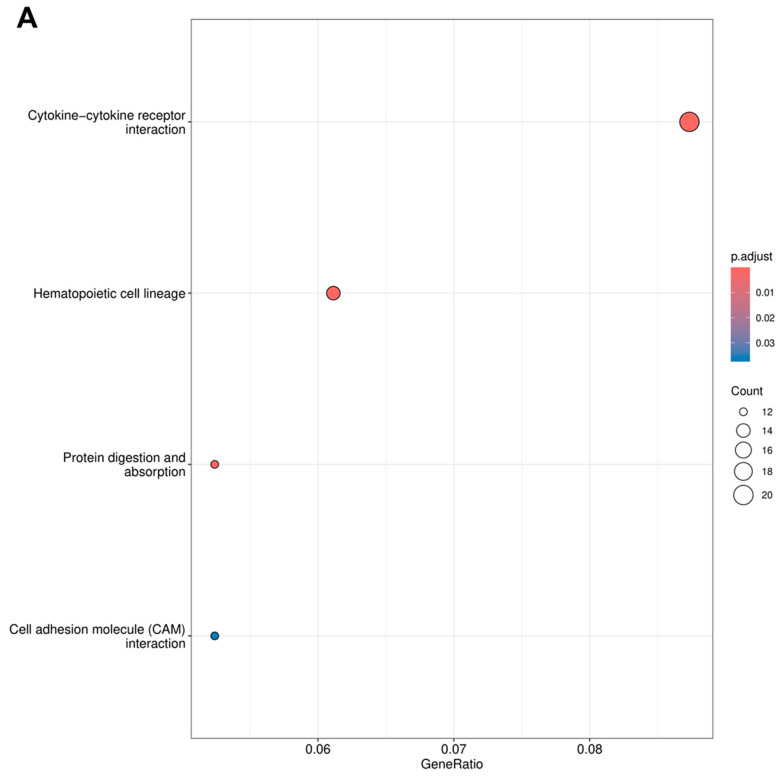

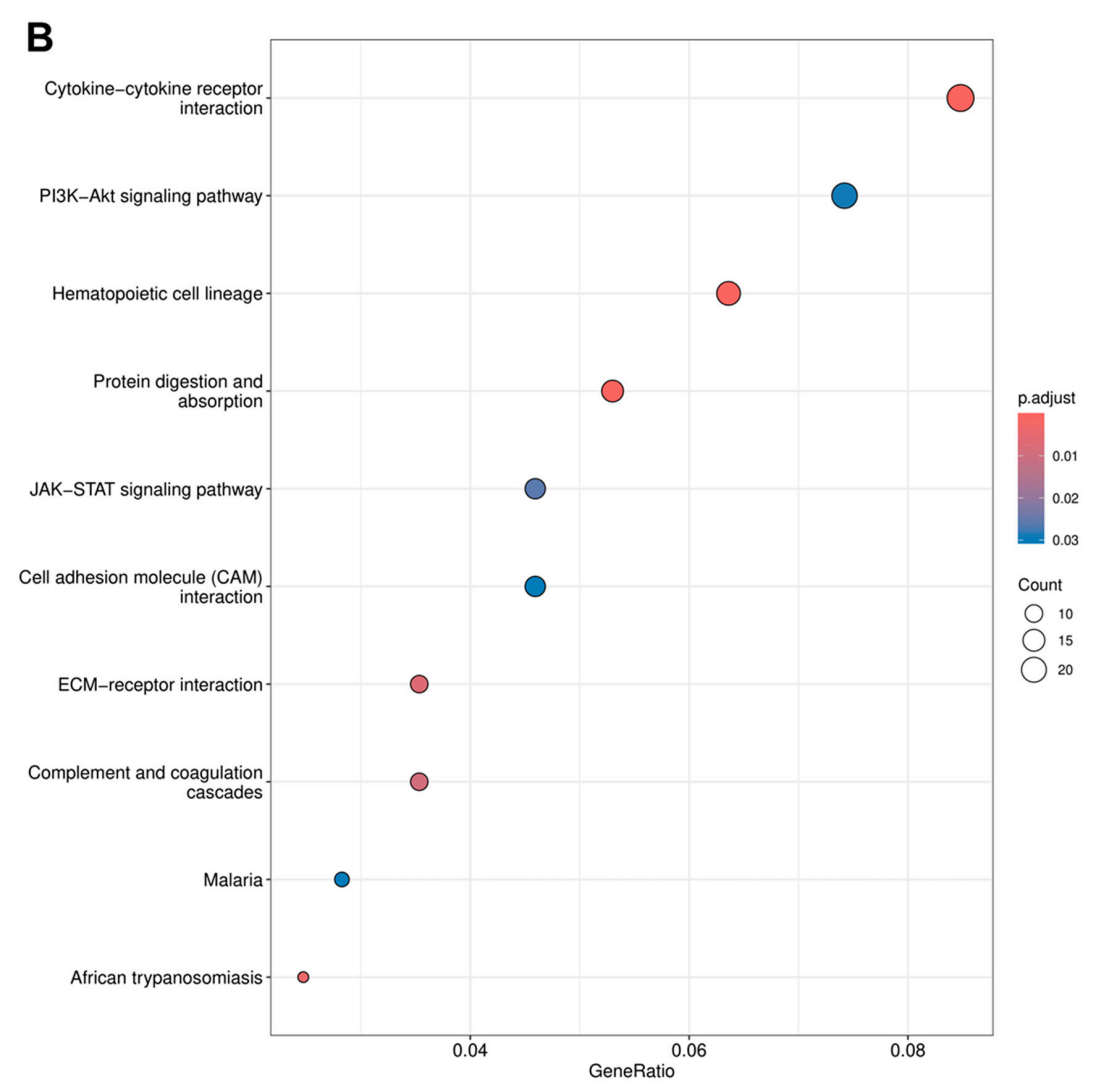
Kyoto Encyclopedia of Genes and Genomes (KEGG) pathway enrichment analysis of DEGs following irradiation. (**A**,**B**) Bubble plots showing significantly enriched KEGG pathways based on DEGs identified by limma-voom (**A**) and DESeq2 (**B**). The *x*-axis represents the GeneRatio, while bubble size corresponds to the number of genes mapped to each pathway. Color intensity indicates the adjusted *p*-value (padj), reflecting enrichment significance. Across both analytical approaches, cytokine–cytokine receptor interaction and hematopoietic cell lineage pathways emerged as dominant features, with additional enrichment of key signaling pathways, including JAK–STAT, PI3K–Akt, extracellular matrix–receptor interaction, and complement and coagulation cascades, highlighting coordinated inflammatory, hematopoietic, and microenvironmental responses to irradiation.

To further clarify the interpretation, KEGG analysis was also performed, showing that DEGs were enriched in the cytokine–cytokine receptor interaction and hematopoietic cell lineage pathways, confirming the link between transcriptomic changes and cytokine signal regulation and hematopoietic dynamics (Figure 3). DESeq2 results also revealed signaling pathways, such as the JAK–STAT and PI3K–Akt pathways, which are well known for cytokine signal transduction, stress responses, and regulation of cell proliferation/survival (Table S5). Furthermore, complement and coagulation cascades were detected, consistent with the enrichment of GO terms related to hemostasis/coagulation, suggesting a link to the systemic response to tissue damage. Other pathways, such as ECM–receptor interaction and cell adhesion molecules (CAMs), corroborate the finding that post-irradiation changes also involve cell–matrix interactions and remodeling of the tissue microenvironment. To complement the DEG-based analysis, we also performed pre-ranked GSEA using the Hallmark collection to evaluate threshold-free pathway changes across the entire transcriptome. GSEA results showed activated enrichment in pathways related to mTORC1 signaling, unfolded protein response, IL6–JAK–STAT3 signaling, PI3K–AKT–mTOR signaling, oxidative phosphorylation, and glycolysis, while pathways such as heme metabolism, interferon-alpha response, TNF-α signaling via NF-κB, and epithelial–mesenchymal transition showed suppressed enrichment (Table S6 and Figure S2). Overall, GO, KEGG, and GSEA results consistently indicate that radiation exposure is associated with coordinated changes in the immune/inflammatory program, hematopoietic function, metabolic stress response, and extracellular matrix dynamics and the tissue microenvironment, which together may contribute to the tissue response to radiation damage.

### 3.4. Analysis of Protein–Protein Interaction Network and Hub Gene Modules

**Figure 4:**
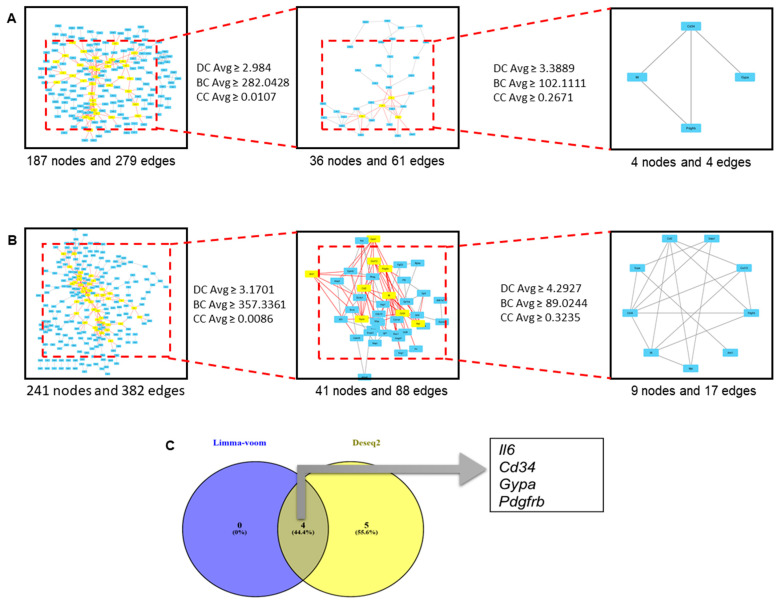
Protein–protein interaction (PPI) network analysis and identification of consensus hub genes following irradiation. (**A**) PPI network constructed from DEGs identified by limma-voom analysis. The full interaction network (**left**) was progressively refined to a highly connected sub-network (middle) and a core module (**right**) based on network topology parameters. Details of the PPI processing results for the limma-voom group are presented in Table S7. (**B**) PPI network con-structed from DEGs identified by DESeq2 analysis, showing the full network (**left**), a refined sub-network (**middle**), and a core hub module (**right**). Hub candidates were selected using CytoNCA based on centrality measures, including degree, betweenness, and closeness. Details of the PPI processing results for the DESeq2 group are presented in Table S8. In the network panels, yellow nodes indicate genes retained after PPI selection. Edges represent interactions in the PPI network, and the dashed red boxes indicate the regions enlarged in the subsequent panels (**C**) Venn diagram illustrating the overlap of hub gene candidates identified by limma-voom and DESeq2 analyses. Four consensus hub genes (Il6, Cd34, Gypa, and Pdgfrb) were consistently identified by both approaches and priori-tized as key molecular regulators linking inflammatory signaling, hematopoietic regulation, erythroid dynamics, and microenvironmental remodeling after irradiation.

To explore the functional relationships among gene products identified as DEGs, PPI network analysis was performed using DEG lists from two statistical approaches (limma-voom and DESeq2). In the limma-voom-based analysis, the initial PPI network comprised 187 nodes and 279 edges, which was then narrowed to a more focused subnetwork (36 nodes and 61 edges) to yield a small core module (4 nodes and 4 edges) (Figure 4A and Table S7). In the DESeq2-based analysis, the initial PPI network was larger (241 nodes and 382 edges), which was subsequently focused into subnetworks (41 nodes and 88 edges) and a core module (9 nodes and 17 edges) (Figure 4B and Table S8). The difference in network size between the two methods is consistent with the finding that DESeq2 identifies more DEGs, leading to more genes that can be mapped to the PPI network. To increase the robustness of the findings, a comparison of candidate genes from both approaches was performed. The Venn diagram shows four genes that consistently appear in both methods: *Il6*, *Cd34*, *Gypa*, and *Pdgfrb* (Figure 4C). All four genes showed the same direction of change in the DEG analysis and were thus prioritized as hub candidate genes for further analysis. Biologically, these candidate genes are related to important functions in the cytokine/inflammatory response (*Il6*), hematopoietic homeostasis and differentiation (*Cd34* and *Gypa*), and niche/microenvironmental regulation through growth and stromal signals (*Pdgfrb*). These findings support the idea that radiation exposure is associated with changes in regulatory networks involving components of the immune–cytokine and hematopoietic systems.

Finally, to further strengthen the identification of hub genes, we performed additional validation analyses using the CytoHubba plugin on the PPI network constructed from significant DEGs identified by limma-voom and DESeq2 (Figures S3 and S4 and Tables S9 and S10). Initially, candidate hub genes were identified using CytoNCA based on primary centrality measures to quantify each node’s topological importance in the network. However, because hub gene selection relying solely on a single centrality metric can potentially introduce methodological bias, we further applied several ranking algorithms available in CytoHubba as a complementary validation approach. This approach allowed for a more comprehensive evaluation of the stability of node rankings across different centrality definitions and network topologies. The results show that *Il6*, *Cd34*, *Gypa*, and *Pdgfrb* remain consistently identified across both pipelines and across multiple topology algorithms. This consistency suggests that these four genes are stable core nodes in the network interactions, not simply the result of selection influenced by a single analytical method. Thus, the combined use of CytoNCA for initial identification and CytoHubba for multi-algorithm validation provides a stronger, more robust, and more reproducible basis for hub gene identification. These findings also reduce the likelihood that prioritized hub gene candidates are simply artifacts of a single centrality metric or of specific biases in any one differential expression analysis pipeline. Conversely, the consistent identification of *Il6*, *Cd34*, *Gypa*, and *Pdgfrb* across multiple analytical approaches suggests that they may represent candidate regulatory genes involved in the hematopoietic response to irradiation.

## 4. Discussion

BM is highly sensitive to radiation, leading to loss of hematopoietic cells and deficiencies of essential white blood cells, platelets, and red blood cells [1]. Concurrently, the ability of HSCs to regenerate by activating self-renewal pathways, as well as to proliferate and differentiate in response to BM tissue damage, depends on intrinsic and extrinsic mechanisms [1,34]. According to the extrinsic mechanism, HSCs are thought to reside within the HSC niche, where supporting cells produce membrane-bound and secreted factors to regulate HSC biological processes (maintenance, differentiation, and self-renewal) [35]. In this study, to gain a more comprehensive understanding of the molecular response after irradiation, the study applied a bioinformatics-based integrative approach to identify gene hubs of DEGs, the biological pathways involved, and prospective therapeutic target candidates. The analysis was performed using RNA-Seq data, a high-throughput sequencing technology, to identify DEG sets and analyze IR injury-related pathways.

Using both analytical pathways in this study provides comprehensive multidimensional information on the molecular dynamics underlying radiation injury. Starting with GO analysis, the BP term is dominated by processes related to the immune response, including adaptive immune response based on somatic recombination of immune receptors with immunoglobulin superfamily domains, lymphocyte-mediated immunity, immunoglobulin-mediated immune response, B cell-mediated immunity, and lymphocyte-mediated immunity. This pattern indicates that radiation exposure triggers amplification of immune-inflammatory signals and intense intercellular communication via immune mediators [36,37], often leading to an inflammatory response in the BM [38,39]. Simultaneously, enrichment was found in myeloid cell homeostasis and erythrocyte development. This suggests a myeloid differentiation bias in hematopoiesis following acute exposure to a stress adaptation mechanism that disrupts hematopoietic balance [40]. The effects of this imbalance were significantly negatively correlated with dose and time, and with the number of nuclear cells in the BM [41]. In the CC domain, terms such as collagen-containing extracellular matrix (ECM) and collagen trimer/complex are enriched, where ECM involvement includes regulating cell adhesion and migration, controlling proliferation and differentiation, and determining cell shape, all of which are important in the stem cell niche [42]. Finally, the MF domain also captures information on antigen binding, immune receptor activity, and cytokine receptor activity. These findings reinforce the notion that radiation responses are highly dependent on activation of the immune–cytokine axis [43].

In line with the GO findings, KEGG pathway analysis revealed the involvement of immune system modules, as well as pathways related to hematopoiesis and cell–microenvironment interactions. The hierarchical relationships among KEGG categories, subcategories, and enriched pathways are further visualized using Sankey diagrams (Figures S5 and S6). In addition, further GSEA showed that irradiation induces a coordinated shift towards stress-adaptive and metabolic pathways while suppressing immune and hematopoietic programs, strengthening the idea of a multilayered transcriptional reprogramming in BM stem cells (Figure S2). One of the most prominent pathways was cytokine–cytokine receptor interaction, consistent with enrichment in MF categories such as cytokine receptor activity. This convergence of findings indicates that genes involved in cytokine signaling play a role in the post-irradiation response in BM, potentially reflecting the activation of inflammatory mediators and tissue stress response pathways [36].

Furthermore, enrichment of the hematopoietic cell lineage pathway suggests that the identified DEGs may reflect perturbations in differentiation programs and hematopoietic homeostasis in response to radiation exposure [44]. Additionally, pathways such as JAK/STAT and PI3K–Akt were enriched in the present study. The JAK/STAT pathway is central to extracellular cytokine receptor-mediated signal transduction and is involved in cell proliferation, differentiation, organ development, and immune homeostasis [45]. In contrast, the PI3K–Akt pathway is a key regulator of inflammatory responses and cytokine release from macrophages [46]. Although several pathways identified in the present analysis, including cytokine signaling and JAK–STAT activation, have been previously implicated in radiation responses, the integrative analytical framework employed here enables identification of consensus hub genes positioned within these pathways. Such network-level prioritization may help highlight candidate regulatory nodes that coordinate inflammatory signaling, hematopoietic regulation, and microenvironmental remodeling following irradiation.

Overall, these pathways overlap with the four identified hub genes. Starting with *Il6*, which aligns with the predominance of cytokine pathways, particularly cytokine–cytokine receptor interaction and associated signal transduction pathways. IL-6 is a pleiotropic cytokine that plays a key role in regulating immunity and inflammation and is also linked to hematopoiesis [47]. Furthermore, IL-6/IL-6R signaling has been reported to contribute to HSC formation and emergence during embryogenesis; in some models, the source of IL-6 is primarily non-HSCs, such as myeloid cells [48]. In adult systems and in vitro cultures, IL-6 is also frequently used (along with other hematopoietic factors) to support HSPC proliferation/expansion, and *Il6* deficiency has been associated with impaired stem/progenitor regulation in some models [48]. Genetic loss of Il6 has been reported to reduce the pool of early progenitors (e.g., CFU-S and pre-CFU-S) and decrease long-term repopulation capacity upon serial transplantation; *Il6* deficiency can also disrupt the balance of committed progenitors (GM, megakaryocytic, and erythroid) and inhibit hematopoietic recovery after myeloablation stress or hemolytic anemia [49]. In this study, decreased *Il6* was observed in the irradiated group compared with the control, consistent with transcriptional suppression associated with injury to the HSC compartment. Since IL-6 is often produced by non-HSCs in the hematopoietic system/microenvironment, this finding does not directly imply that overall IL-6 levels in the BM are decreased. Previous animal studies have shown that exogenous IL-6 administration after irradiation can accelerate hematopoietic recovery; thus, the IL-6 axis can be viewed as a testable therapeutic hypothesis that requires specific validation regarding timing of administration, dosage, and safety [50]. For example, in a model of hematopoietic depression induced by sublethal irradiation, exogenous IL-6 was reported to accelerate the recovery of multilineage hematopoiesis, as evidenced by accelerated regeneration of CFU-S, GM-CFC, and CFU-E, and recovery of peripheral blood cells [50].

Furthermore, *Cd34* is a macromolecular transmembrane sialomucin protein; related family members include podocalyxin and endoglycan. *Cd34* has long been used as a key clinical marker for the identification and purification of primitive HSPCs [51,52]. At the level of homing mechanisms, studies in humans and mice have shown that E-selectin and P-selectin are constitutively expressed on BM endothelial cells, and intravital studies have revealed that HSPC migration/homing to the BM occurs within a specialized microvascular network where E-selectin is expressed [51]. Similarly, approaches based on P-selectin-coated devices have been reported to enhance CD34^+^ HSPC engraftment compared to anti-CD34 antibody-coated devices, highlighting the importance of P-selectin interactions in early HSPC engraftment [53,54]. Transplant studies in recipients exposed to lethal radiation have shown that the loss of both endothelial selectins (E- and P-selectin) disrupts the early stages of HSPC interaction/adhesion with the BM microvasculature, resulting in decreased homing and decreased post-transplant survival (most pronounced at a limited donor cell dose). In contrast, when either selectin is present, homing and hematopoietic reconstitution are relatively intact [55].

However, it is important to emphasize that, in the murine system, *Cd34* expression on HSC is dynamic and reflects activation status rather than a static attribute [56]. Murine transplant studies have shown that the majority of cells with long-term engraftment capability in normal adult mice are in the CD34− fraction, but after stress/activation (e.g., following 5-FU), stem cells can be found in both the CD34− and CD34+ fractions. Both in vivo and in vitro activation are associated with the appearance of CD34 expression and can then revert to the CD34− state when the BM returns to steady state [56]. Furthermore, in the G-CSF mobilization model, most mobilized stem cells express CD34. However, when the cells return to the BM, and the BM recovers from radiation-induced hypoplasia and reaches a steady state, CD34 expression is reported to decrease to undetectable levels [57].

Furthermore, the contribution of Cd34 extends beyond mere markers to include adhesion and transmigration dynamics. In transplantation assays, Cd34−/− and wild-type HSCs have been reported to exhibit relatively equivalent engraftment abilities in lethally irradiated recipients. However, when transplanted into sublethally irradiated or unirradiated animals, the engraftment capacity of Cd34−/− HSCs was reduced by less than 20% compared to wild-type [58,59,60]. This conditioning-dependent difference supports the hypothesis that Cd34 (as sialomucin) acts as an anti-adhesive sialomucin layer that helps HSCs extravasate across the endothelium into the BM niche. Conversely, irradiation that causes inflammation and increases vascular permeability may reduce the relative need for this anti-adhesive function, so that the Cd34−/− defect appears more subtle under lethal irradiation conditions [60,61]. Furthermore, recent single-cell transcriptomic studies have further refined the understanding of hematopoietic stem cell heterogeneity and the dynamic expression patterns of canonical markers such as CD34 during stress-induced hematopoietic remodeling [62]. These studies highlight that HSC marker expression may fluctuate depending on activation state and microenvironmental cues.

In addition to *Il6* and *Cd34*, the *Gypa* and *Pdgfrb* genes were also consistently identified as candidate hub genes in this study. Biologically, *Gypa* (glycophorin A) is one of the major sialoglycoproteins on the erythroid cell membrane and is widely used as an erythroid marker in both humans and mice [63,64]. In the context of radiation injury, several studies have shown that radiation exposure can rapidly reduce erythroid progenitors and precursors in the early phase, accompanied by activation of apoptotic mechanisms and changes in the dynamics of erythropoiesis recovery. On the other hand, the PDGF–PDGFR axis, including PDGFRβ in the stromal/perivascular context, has been associated with the activation of pro-survival pathways, such as PI3K/Akt, and with microenvironmental remodeling after irradiation [65,66,67]. Because the RNA-seq data were generated from sorted HSC populations, the detection of transcripts typically associated with erythroid (*Gypa*) or stromal (*Pdgfrb*) lineages may reflect minor contamination or microenvironment-derived transcriptional signals rather than direct lineage specification within HSCs. Alternatively, radiation-induced alterations in cell composition or transcriptional spillover from closely interacting niche cells may contribute to the observed expression patterns.

Overall, this study aimed to investigate molecular changes associated with radiation-induced hematopoietic injury in the BM through an integrative bioinformatics analysis of RNA-sequencing data obtained from murine BM-derived HSCs following exposure to IR (5 Gy). To improve analytical robustness, two independent differential expression analysis approaches, limma-voom and DESeq2, were applied in parallel, and the resulting DEGs were systematically examined through GO and KEGG enrichment analyses, as well as PPI network construction. This integrative framework revealed significant alterations in biological pathways related to cytokine signaling, hematopoietic lineage regulation, erythroid-associated processes, and microenvironmental regulation at the HSC transcriptomic level following irradiation. Notably, network centrality analysis consistently identified four consensus hub genes (*Il6*, *Cd34*, *Gypa*, and *Pdgfrb)* across both analytical pipelines, all of which were significantly downregulated after radiation exposure. The coordinated downregulation of these genes may reflect radiation-associated transcriptional perturbation within the HSC compartment and/or changes in hematopoietic or niche-related signals detected in the sorted cell population.

Importantly, the RNA-seq dataset analyzed in this study represents a single time point (day 3) following irradiation at a single radiation dose (5 Gy). Because radiation-induced hematopoietic injury and recovery are dynamic, multi-phasic processes involving acute cellular damage, transient regenerative signaling, and longer-term niche remodeling, the transcriptional signatures identified here are more appropriately interpreted as reflecting early radiation-response processes rather than the full temporal trajectory of hematopoietic recovery. In addition, an alternative explanation for the observed differential expression patterns is that they may partially reflect shifts in cellular composition within the sorted HSC population following irradiation. Radiation-induced stress may alter the relative abundance of closely associated progenitor or niche-derived cell types, thereby influencing the transcriptomic signals detected in this bulk RNA-seq dataset. Therefore, the identified hub genes should be interpreted as candidate regulatory markers associated with radiation-induced BM injury, rather than definitive drivers of injury or validated biomarkers.

By employing a computational strategy independent of a single analytical method, this study provides a systems-level perspective on the molecular axes potentially associated with radiation-induced hematopoietic injury and prioritizes candidate genes as molecular reference points for further research on radiation injury assessment. However, a major limitation of this study is the lack of independent experimental validation of the identified key genes. Therefore, further research involving approaches such as RT-qPCR, protein expression analysis, and functional perturbation experiments is needed to confirm the biological relevance and functional roles of these candidate genes. Furthermore, the findings of this study can serve as a foundation for further studies in the field of biodosimetry and to deepen our understanding of the mechanisms of hematopoietic damage and recovery after radiation exposure. Future integrative analyses incorporating transcription factor inference approaches, such as DoRothEA or TRRUST, could also help identify the upstream regulatory networks underlying the decreased expression of the candidate hub genes identified in this study.

## 5. Conclusions

This study used RNA-seq data to identify DEGs, perform functional enrichment analysis, and prioritize hub genes in BM after radiation exposure. Overall, the analysis results indicate that radiation-induced hematopoietic injury is associated with disruption of cytokine signaling pathways and immunological responses, particularly the cytokine–cytokine receptor interaction pathway, including JAK/STAT and PI3K-Akt. Furthermore, the enrichment of terms related to hematopoiesis and the extracellular matrix suggests that microenvironmental or niche dynamics, tissue remodeling, and changes in the hematopoietic compartment likely contribute to the biological response after radiation. The hub genes identified in this study represent complementary biological axes in the regulation of inflammatory signaling, hematopoietic homeostasis, and cell–niche interactions. These findings support the idea that the BM response to radiation involves not only acute inflammatory activation but also changes in the hematopoietic program and its supporting microenvironment. However, because this study is based on computational analysis of a single public dataset, these genes are more appropriately considered candidate regulators or transcriptomic indicators potentially associated with radiation-induced hematopoietic injury, rather than definitive validated molecular targets. Therefore, further experimental validation is needed to confirm the biological relevance and translational potential of these findings.

## Figures and Tables

**Table 1:** Summary of RNA-seq read alignment statistics for BM samples following irradiation. Total reads, mapped reads, uniquely mapped reads, multi-mapped reads, unmapped reads, and overall alignment rates (%) were obtained by mapping high-quality RNA-seq reads to the reference mouse genome using HISAT2.

Samples	Total Reads	Mapped Reads	Uniquely Mapped Reads	Multi-Mapped Reads	Unmapped Reads	Alignment Rate (%)
SRR26338106	21,834,855	19,670,550	18,590,829	1,079,721	2,164,305	90.09
SRR26338107	21,406,072	19,677,109	18,509,378	1,167,731	1,728,963	91.92
SRR26338108	21,500,952	20,237,108	19,176,351	1,060,757	1,263,844	94.12
SRR26338102	22,605,349	20,367,425	19,324,231	1,043,194	2,237,924	90.10
SRR26338103	22,078,482	19,898,679	18,887,834	1,010,845	2,179,803	90.13
SRR26338105	23,691,957	21,373,325	20,276,244	1,097,081	2,318,632	90.21

## Data Availability

All data and information related to this study are fully presented in the main text and Supplementary Materials.

## Supplementary Materials

The following supporting information can be downloaded at: https://www.mdpi.com/article/10.3390/genes17040377/s1, Figure S1. Diagnostic quality control of RNA-seq analysis on irradiated (IR) and control (Ctrl) mouse bone marrow hematopoietic stem cells. (A) Voom mean–variance trend plot showing the relationship between expression level and variance prior to limma-based differential expression analysis. The red curved line represents the voom trend line, indicating that the mean–variance relationship was appropriately modeled for downstream linear modeling. (B) DESeq2 dispersion estimation plot showing gene-wise dispersion estimates (black), the fitted dispersion trend (red), and the final dispersion estimates after shrinkage (blue). The expected inverse relationship between mean expression and dispersion supports stable variance estimation for differential expression analysis. (C) Histogram of raw *p*-values for the comparison between IR and Ctrl samples. The enrichment of *p*-values near zero is consistent with the presence of genuine differential expression signals rather than random noise, and (D) MA plot showing log fold change against the mean of normalized counts; blue dots indicate significantly differentially expressed genes (padj < 0.05). The overall symmetric distribution around zero suggests appropriate normalization and no obvious systematic bias in fold-change estimation. Figure S2. GSEA of Hallmark pathways. Bubble plot showing the top 15 enriched Hallmark gene sets ranked by normalized enrichment score; Figure S3. CytoHubba analysis of the protein–protein interaction (PPI) network constructed from DEGs identified using limma-voom approach; Figure S4. CytoHubba analysis of the protein–protein interaction (PPI) network constructed from DEGs identified using DESeq2 approach; Figure S5. Sankey diagram illustrating KEGG pathway enrichment based on limma-voom analysis. The Sankey plot visualizes the hierarchical relationships among KEGG functional categories, subcategories, and the top 10 significantly enriched KEGG pathways derived from differentially expressed genes (DEGs). The width of each flow represents the relative number of genes contributing to each pathway, highlighting the dominant involvement of signaling molecules and interaction, immune system–related pathways, and organismal system processes in the transcriptional response to irradiation; Figure S6. Sankey diagram illustrating KEGG pathway enrichment based on DESeq2 analysis. The Sankey plot depicts the hierarchical relationships among KEGG functional categories, subcategories, and the top 10 significantly enriched KEGG pathways derived from differentially expressed genes (DEGs) identified by DESeq2. The width of each flow corresponds to the relative number of genes contributing to each pathway, highlighting prominent enrichment of signaling transduction, immune system–related processes, and disease-associated pathways, including cytokine–cytokine receptor interaction, JAK–STAT and PI3K–Akt signaling, hematopoietic cell lineage, and complement and coagulation cascades in response to irradiation. Table S1. Summary of sequencing read-quality metrics before and after quality filtering and adapter trimming using fastp; Table S2. DEGs results based on limma-voom analysis; Table S3. DEGs results based on DESeq2 analysis; Table S4. GO results based on limma-voom and DESeq2 analysis; Table S5. KEGG results based on limma-voom and DESeq2 analysis; Table S6. GSEA of Hallmark pathways output; Table S7. PPI Process Results of limma-voom group by CytoNCA; Table S8. PPI Process Results of DESeq2 group by CytoNCA; Table S9. List of the genes present from twelve different methods of the CytoHubba analysis based on the limma-voom approach; and Table S10. List of the genes present from twelve different methods of the CytoHubba analysis based on the DESeq2 approach.

## Abbreviations

The following abbreviations are used in this manuscript:

IR	Ionizing radiation
HSCs	Hematopoietic stem cells
HPCs	Hematopoietic progenitor cells
TBI	Total body irradiation
PPI	Protein–protein interaction
DEGs	Differentially expressed genes
BM	Bone marrow
GO	Gene ontology
BP	Biological process
CC	Cellular component
MF	Molecular function
KEGG	Kyoto encyclopedia of genes and genomes
GSEA	Gene set enrichment analysis
log_2_FC	log_2_fold change
