# Supplementary material for: Identification of Radiation-Induced Injury Pathways and Hub Genes from RNA-Seq Data Based on Integrative Bioinformatics Approach
Source: Genes (Basel). 2026 Mar 27;17(4):377. doi: 10.3390/genes17040377 (PMC13116960; doi:10.3390/genes17040377)
Supplement: Supplementary file 1 [file genes-17-00377-s001.zip › genes-4208701-supplementary.pdf]

Identification of Radiation-Induced Injury Pathways and Hub Genes from RNA-Seq Data Based on Integrative Bioinformatics Approach

Supplementary Tables & Figures (with captions)

Khalish Arsy Al Khairy Siregar [1], Chi-Ho Lee [1], Jong-Jin Kim [2], Dong-Jo Chang [1,3] and Seung-Hyun Jeong [1,3,*]

1 College of Pharmacy, Sunchon National University, 255 Jungang-ro, Suncheon-si 57922, Jeollanam-do, Republic of Korea

2 Department of Biomedical Science, Sunchon National University, Suncheon-si 57922, Jeollanam-do, Republic of Korea

3 College of Pharmacy and Research Institute of Life and Pharmaceutical Sciences, Sunchon National University, Suncheon-si 57922, Jeollanam-do, Republic of Korea

* Correspondence: jeongsh@scnu.ac.kr

Table S1. Summary of sequencing read-quality metrics before and after quality filtering and adapter trimming using fastp.

SRR Code	Before filtering				After filtering				Adapter Content (%)
	total reads (M)	Q20 bases (G)	Q30 bases (G)	GC (%)	total reads (M)	Q20 bases (G)	Q30 bases (G)	GC (%)	
SRR26338106	44.015560	6.351743 (96.97%)	6.019129 (91.89%)	49.54	43.669710	6.311910 (97.23%)	5.987536 (92.24%)	49.54	Read1 = ~0.070<br>Read2 = ~0.067
SRR26338107	43.063858	6.229817 (97.24%)	5.920914 (92.42%)	48.57	42.812144	6.199266 (97.45%)	5.896384 (92.69%)	48.57	Read1 = ~0.075<br>Read2 = ~0.073
SRR26338108	43.464864	6.264050 (96.74%)	5.914884 (91.34%)	48.74	43.001904	6.211447 (97.07%)	5.871730 (91.77%)	48.75	Read1 = ~0.085<br>Read2 = ~0.082
SRR26338102	45.560520	6.581597 (97.08%)	6.247720 (92.15%)	49.75	45.210698	6.541545 (97.34%)	6.215995 (92.50%)	49.76	Read1 = ~0.065<br>Read2 = ~0.063
SRR26338103	44.471414	6.422930 (97.04%)	6.091341 (92.03%)	49.71	44.156964	6.386354 (97.28%)	6.062218 (92.35%)	49.71	Read1 = ~0.065<br>Read2 = ~0.063
SRR26338105	47.715656	6.897271 (97.12%)	6.548160 (92.20%)	49.75	47.383914	6.858703 (97.36%)	6.517453 (92.51%)	49.75	Read1 = ~0.065<br>Read2 = ~0.062

Table S2. DEGs results based on limma-voom analysis

(Full data is available in the Excel file Supplementary Tables S2.)

Table S3. DEGs results based on DESeq2 analysis

(Full data is available in the Excel file Supplementary Tables S3.)

Table S4. GO results based on limma-voom and DESeq2 analysis

(Full data is available in the Excel file Supplementary Tables S4.)

Table S5. KEGG results based on limma-voom and DESeq2 analysis

(Full data is available in the Excel file Supplementary Tables S5.)

Table S6. GSEA of Hallmark pathways output

(Full data is available in the Excel file Supplementary Tables S6.)

Table S7. PPI Process Results of limma-voom group by CytoNCA

(Full data is available in the Excel file Supplementary Tables S7.)

Table S8. PPI Process Results of DESeq2 group by CytoNCA

(Full data is available in the Excel file Supplementary Tables S8.)

Table S9. List of the genes present from twelve different methods of the CytoHubba analysis based on the limma-voom approach

Gene	Present In (Methods)
Gypa	BottleNeck, MCC, MNC, Degree, EPC, EcCentricity, Closeness, Radiality, Betweenness, and Stress
Cd34	BottleNeck, Degree, EPC, EcCentricity, Closeness, Radiality, Betweenness, and Stress
Il6	BottleNeck, MNC, Degree, EPC, Closeness, Radiality, Betweenness, and Stress
Pdgfrb	BottleNeck, Degree, Closeness, Radiality, and Betweenness

Table S10. List of the genes present from twelve different methods of the CytoHubba analysis based on the DESeq2 approach

Gene	Present In (Methods)
Gypa	BottleNeck, MCC, MNC, Degree, EPC, EcCentricity, Closeness, Radiality, Betweenness, and Stress

Il6	BottleNeck, MNC, Degree, EPC, EcCentricity, Closeness, Radiality, Betweenness, and Stress
Cd34	BottleNeck, Degree, EPC, Closeness, Radiality, Betweenness, and Stress
Pdgfrb	BottleNeck, Degree, EPC, Closeness, Radiality, Betweenness, and Stress

Supplementary Figure captions

Figure S1. Diagnostic quality control of RNA-seq analysis on irradiated (IR) and control (Ctrl) mouse bone marrow hematopoietic stem cells. (A) Voom mean–variance trend plot showing the relationship between expression level and variance prior to limma-based differential expression analysis. The red curved line represents the voom trend line, indicating that the mean–variance relationship was appropriately modeled for downstream linear modeling. (B) DESeq2 dispersion estimation plot showing gene-wise dispersion estimates (black), the fitted dispersion trend (red), and the final dispersion estimates after shrinkage (blue). The expected inverse relationship between mean expression and dispersion supports stable variance estimation for differential expression analysis. (C) Histogram of raw *p*-values for the comparison between IR and Ctrl samples. The enrichment of *p*-values near zero is consistent with the presence of genuine differential expression signals rather than random noise, and (D) MA plot showing log fold change against the mean of normalized counts; blue dots indicate significantly differentially expressed genes (padj < 0.05). The overall symmetric distribution around zero suggests appropriate normalization and no obvious systematic bias in fold-change estimation.

Figure S2. GSEA of Hallmark pathways. Bubble plot showing the top 15 enriched Hallmark gene sets ranked by normalized enrichment score

Figure S3. CytoHubba analysis of the protein–protein interaction (PPI) network constructed from DEGs identified using limma-voom approach

Figure S4. CytoHubba analysis of the protein–protein interaction (PPI) network constructed from DEGs identified using DESeq2 approach

Figure S5. Sankey diagram illustrating KEGG pathway enrichment based on limma-voom analysis. The Sankey plot visualizes the hierarchical relationships among KEGG functional categories, subcategories, and the top 10 significantly enriched KEGG pathways derived from differentially expressed genes (DEGs). The width of each flow represents the relative number of genes contributing to each pathway, highlighting the dominant involvement of signaling molecules and interaction, immune system–related pathways, and organismal system processes in the transcriptional response to irradiation.

Figure S6. Sankey diagram illustrating KEGG pathway enrichment based on DESeq2 analysis. The Sankey plot depicts the hierarchical relationships among KEGG functional categories, subcategories, and the top 10 significantly enriched KEGG pathways derived from differentially expressed genes (DEGs) identified by DESeq2. The width of each flow corresponds to the relative number of genes contributing to each pathway, highlighting prominent enrichment of signaling transduction, immune

system–related processes, and disease-associated pathways, including cytokine–cytokine receptor interaction, JAK–STAT and PI3K–Akt signaling, hematopoietic cell lineage, and complement and coagulation cascades in response to irradiation.

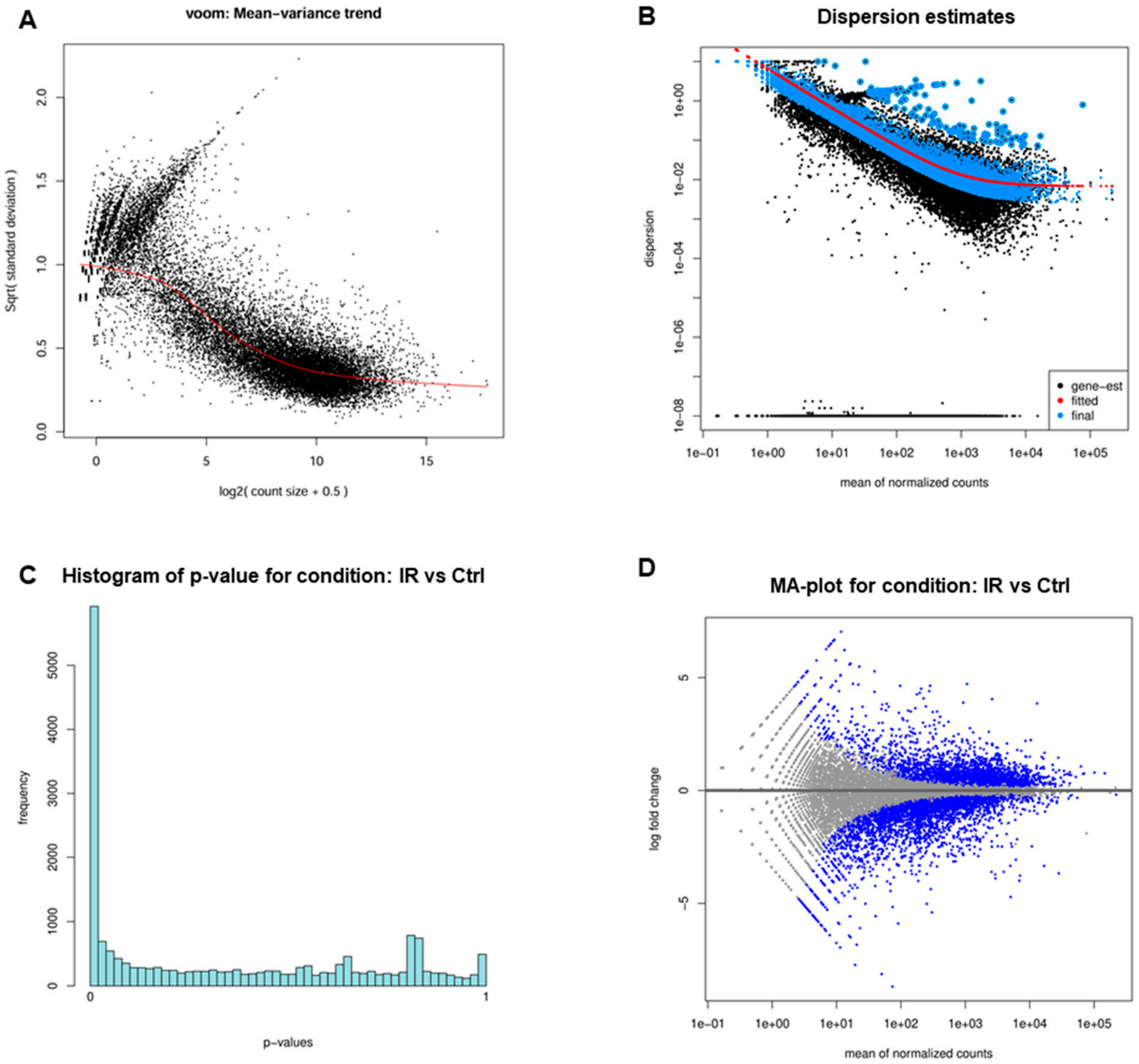
A
voom: Mean−variance trend
Sqrt(standard deviation)
log2(count size + 0.5)
B
Dispersion estimates
dispersion
mean of normalized counts
gene−est
fitted
final
C
Histogram of p-value for condition: IR vs Ctrl
frequency
p−values
D
MA-plot for condition: IR vs Ctrl
log fold change
mean of normalized counts

Figure S1

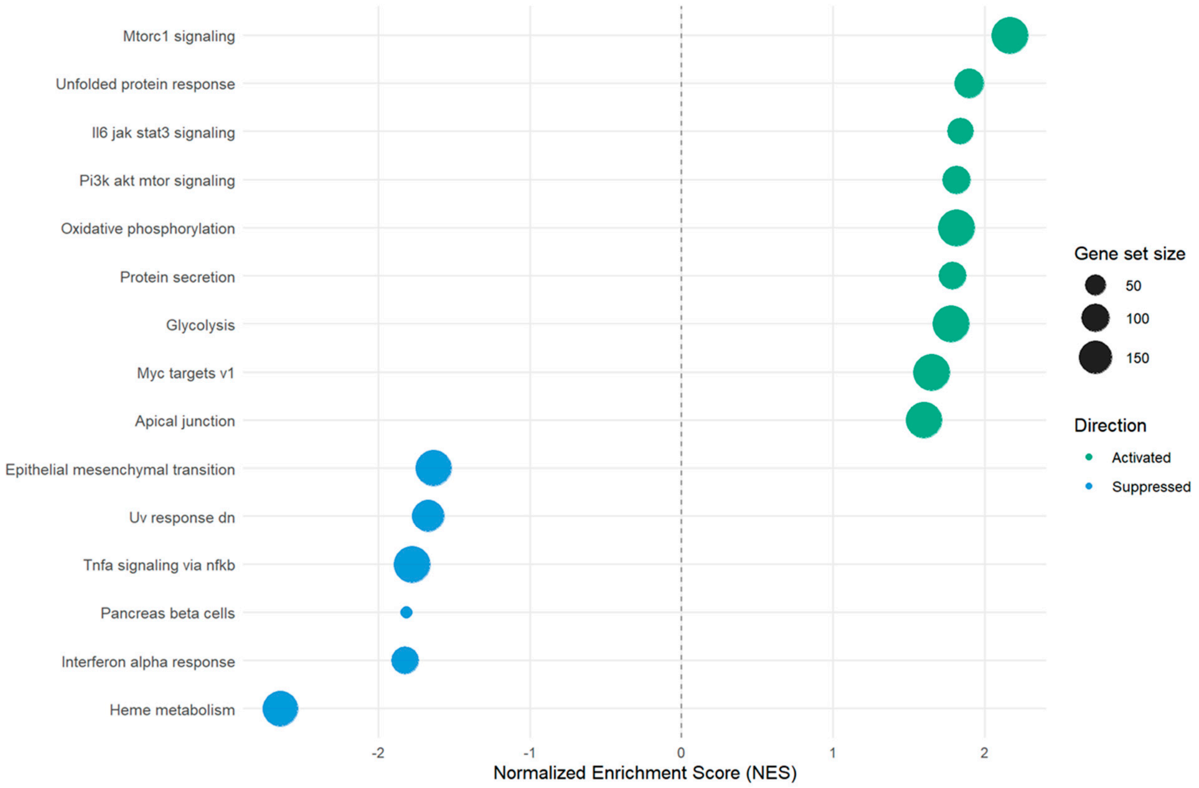
Mtorc1 signaling
Unfolded protein response
Il6 jak stat3 signaling
Pi3k akt mtor signaling
Oxidative phosphorylation
Protein secretion
Glycolysis
Myc targets v1
Apical junction
Epithelial mesenchymal transition
Uv response dn
Tnfa signaling via nfkb
Pancreas beta cells
Interferon alpha response
Heme metabolism
-2
-1
0
1
2
Normalized Enrichment Score (NES)
Gene set size
50
100
150
Direction
Activated
Suppressed

Figure S2

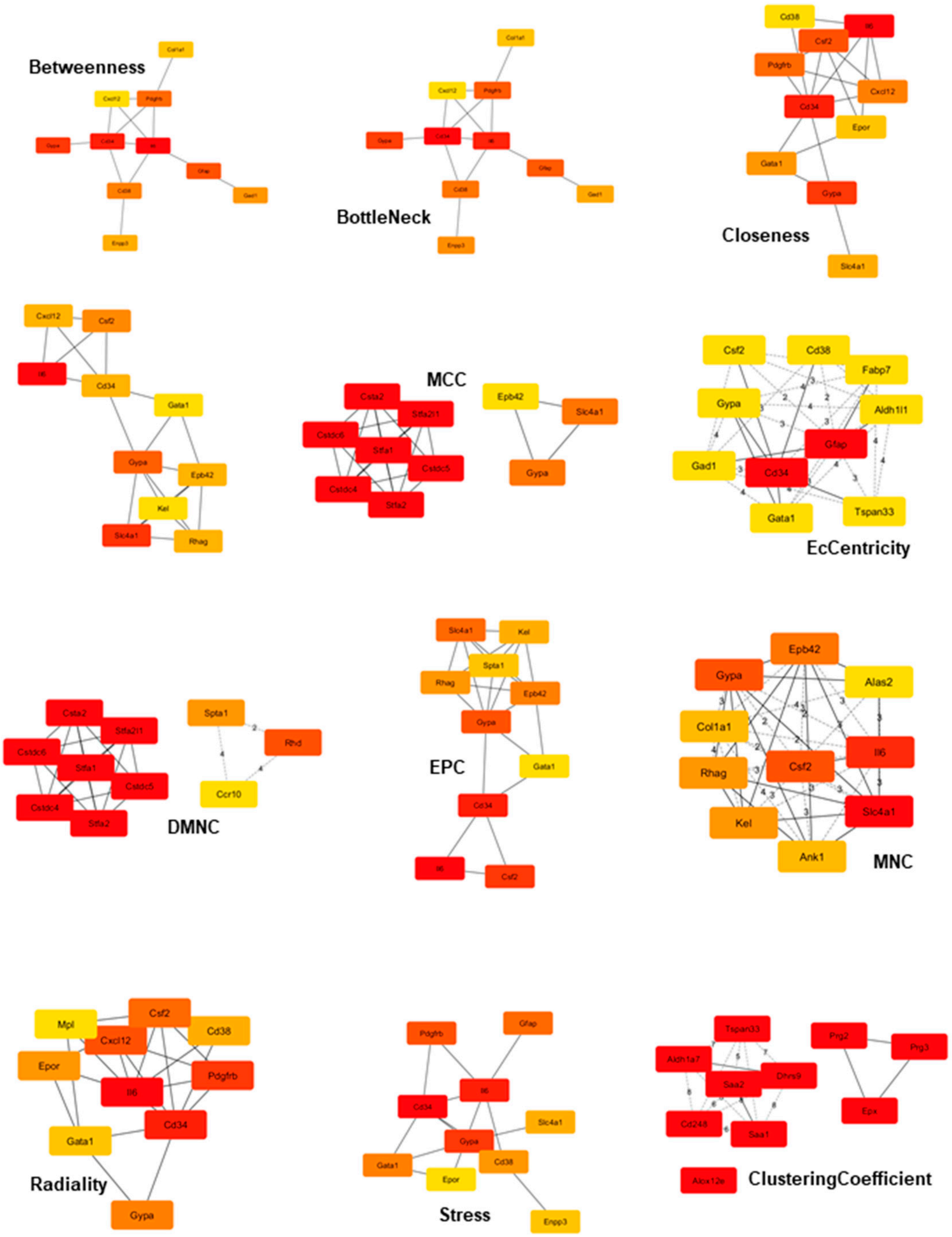
Betweenness
BottleNeck
Closeness
MCC
EcCentricity
DMNC
EPC
MNC
Radiality
Stress
ClusteringCoefficient

Figure S3

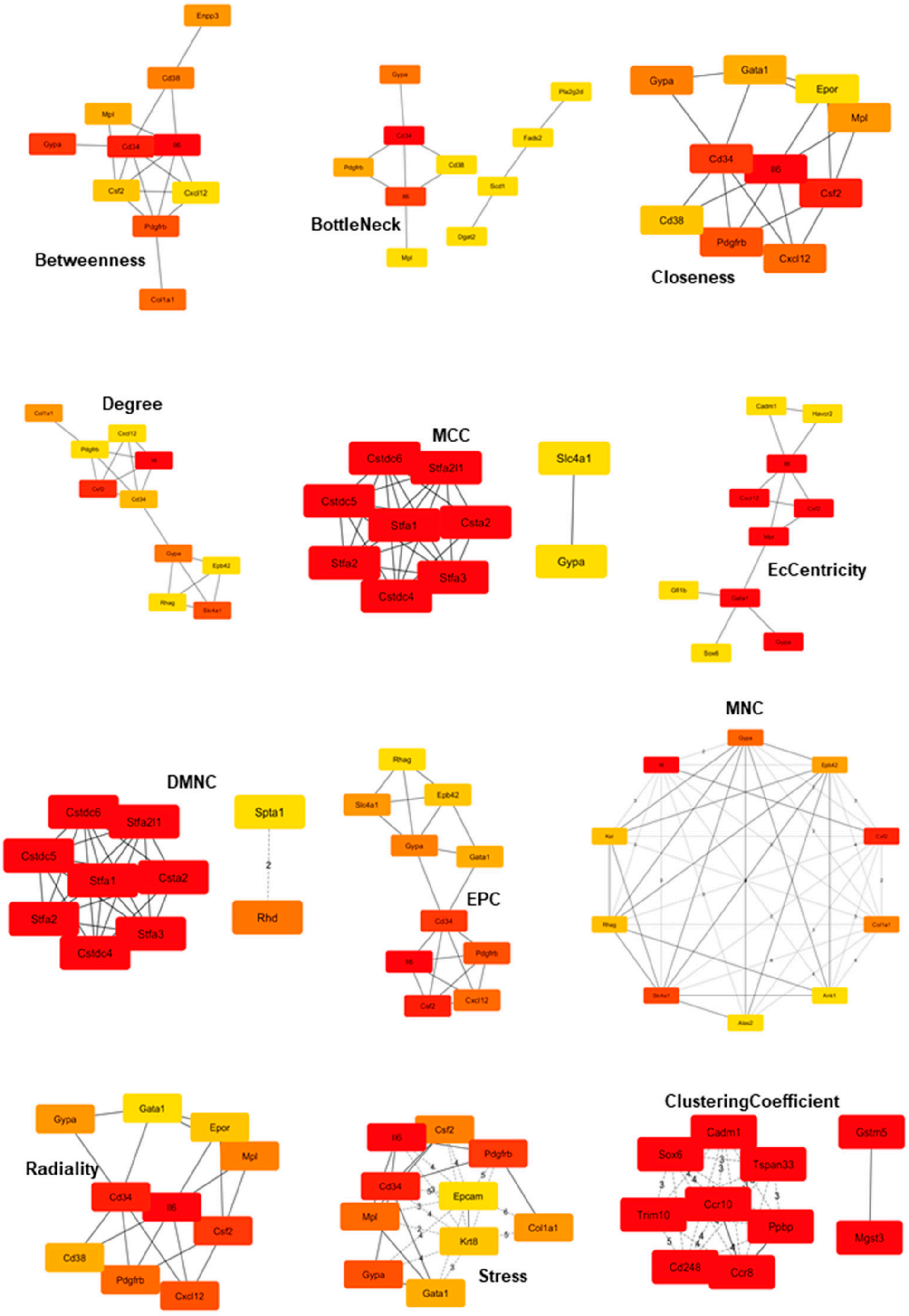
Betweenness
BottleNeck
Closeness
Gypa
Gata1
Epor
Mpl
Cd34
Il6
Csf2
Cd38
Pdgfrb
Cxcl12
Degree
MCC
Cstdc6
Stfa2l1
Cstdc5
Stfa1
Csta2
Stfa2
Stfa3
Cstdc4
Slc4a1
Gypa
EcCentricity
MNC
DMNC
Cstdc6
Stfa2l1
Cstdc5
Stfa1
Csta2
Stfa2
Stfa3
Cstdc4
Spta1
Rhd
EPC
Radiality
Gypa
Gata1
Epor
Mpl
Cd34
Il6
Csf2
Cd38
Pdgfrb
Cxcl12
Stress
Il6
Csf2
Pdgfrb
Cd34
Epcam
Mpl
Krt8
Col1a1
Gypa
Gata1
ClusteringCoefficient
Cadm1
Sox6
Tspan33
Ccr10
Trim10
Ppbp
Cd248
Ccr8
Gstm5
Mgst3

Figure S4

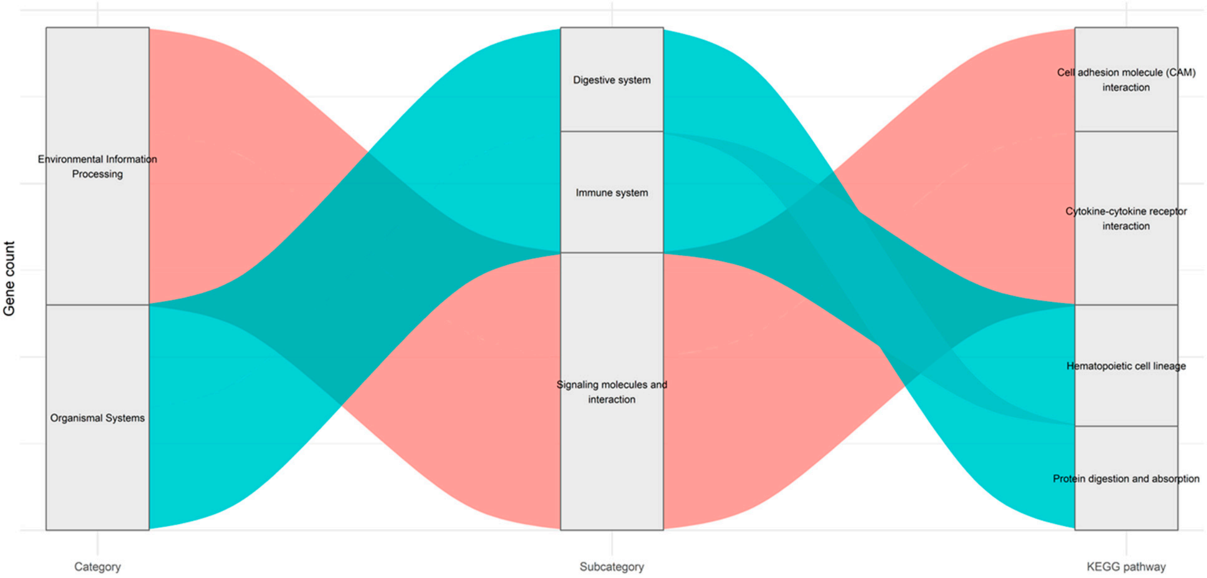

Figure S5

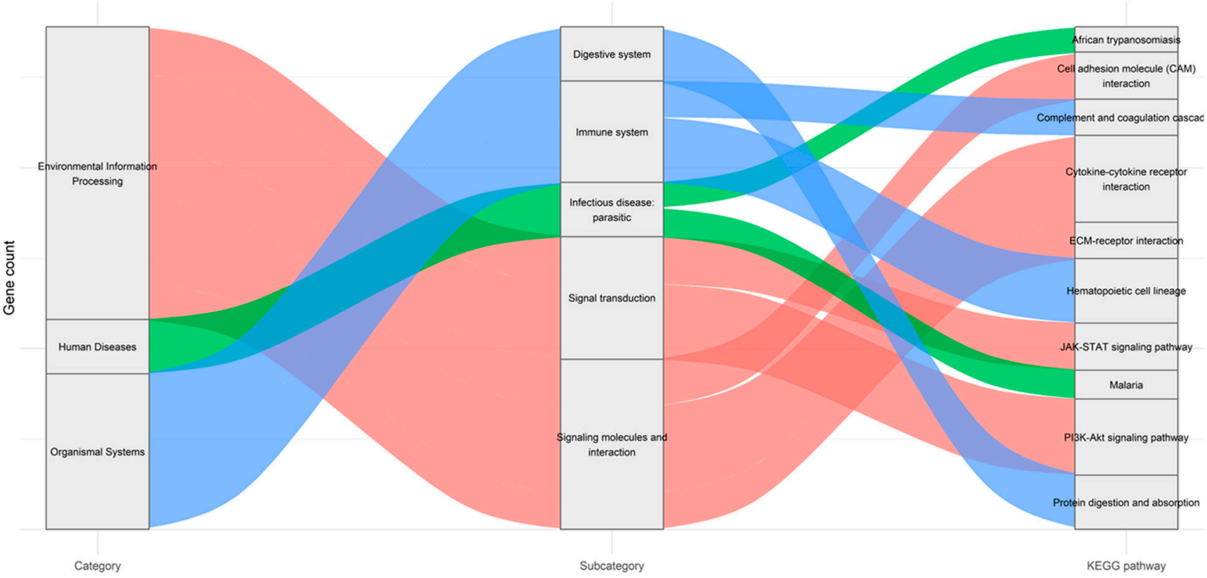

Figure S6
